# Development of a reverse transcription recombinase polymerase based isothermal amplification coupled with lateral flow immunochromatographic assay (CTV-RT-RPA-LFICA) for rapid detection of *Citrus tristeza virus*

**DOI:** 10.1038/s41598-020-77692-w

**Published:** 2020-11-26

**Authors:** Dilip Kumar Ghosh, Sunil B. Kokane, Siddarame Gowda

**Affiliations:** 1 ICAR- Central Citrus Research Institute, Amravati Road, Nagpur, 440 033 India; 2 Citrus Research and Education Center, University of Florida, 700 Experiment Station Road, Lake Alfred, FL 33850 USA

**Keywords:** Virology, Microbiology, Biological techniques

## Abstract

Tristeza is a highly destructive disease of citrus caused by the phloem-limited, flexuous filamentous *Citrus tristeza virus* (CTV) in the genus *Closterovirus* and the family *Closteroviridae.* It has been a major constraint for higher productivity and has destroyed millions of citrus trees globally. CTV is graft transmissible and spread through use of virus infected nursery plants. Therefore, virus detection by using specific and reliable diagnostic tools is very important to mitigate disease outbreaks. Currently, the standard molecular techniques for CTV detection include RT-PCR and RT-qPCR. These diagnostic methods are highly sensitive but time consuming, labor intensive and require sophisticated expensive instruments, thus not suitable for point-of-care use. In the present study, we report the development of a rapid, sensitive, robust, reliable, and highly specific reverse transcription-RPA technique coupled with a lateral flow immunochromatographic assay (CTV-RT-RPA-LFICA). RT-RPA technique was standardized to amplify the coat protein gene of CTV (CTV-p25) and detect double labeled amplicons on a sandwich immunoassay by designing specific labeled primer pair and probe combinations. The optimally performing primer set (CTRPA-F1/CTRPA-R9-Btn) and the corresponding TwistAmp nfo probe (CTRPA-Probe) was optimized for temperature and reaction time using purified cDNA and viral RNA as template. The sensitivity of the developed assay was compared with other detection techniques using in vitro-transcribed RNA. The efficacy and specificity of the assay was evaluated using CTV positive controls, healthy samples, field grown citrus plants of unknown status, and other virus and bacterial pathogens that infect citrus plants. The RT-RPA-LFICA was able to detect ≤ 141 fg of RNA when cDNA used as a template. The assay detected ≤ 0.23 ng/µl of CTV RNA when directly used as template without cross-reactivity with other citrus pathogens. Best results were achieved at the isothermal temperature of 40 °C within 15–20 min. The study demonstrated that RT-RPA-LFICA has potential to become an improved detection technique for end users in bud-wood certification and quarantine programs and a promising platform for rapid point-of-care diagnostics for citrus farmers and small nurseries in low resource settings.

## Introduction

Tristeza is one of the most devastating viral disease of citrus caused by *Citrus tristeza virus* (CTV). It belongs to the genus *Closterovirus*, family *Closteroviridae* and has threatened the citrus industry worldwide by destroying over hundreds of millions of productive trees^1–3^. CTV is one of the most challenging viruses that has induced epidemics of quick decline and has made devastating economic impacts on the citrus industries globally. The most destructive epidemics occurred in Argentina, California, Brazil, Florida, Spain, Israel, and Venezuela^4^. Phloem-limited long flexuous filamentous virions of CTV (2000 × 11 nm) contained single stranded plus sense RNA of approximately 19.3 kb and organized into 12 ORFs (with UTRs at the 5′ and 3′ termini) that potentially encode at least 19 proteins^3–6^. Different aphid species, *Aphis gossypii* Glover, *Aphis* (*Toxoptera*) *citricidus* Kirkaldy and *Aphis spiraecola* Patch act as vectors and transmit CTV from infected to healthy plants in citrus groves in a semi-persistent manner^3,7^. Consequently, reduced production and fruit quality and increase in disease severity result in citrus decline. The symptoms of Tristeza (stem pitting, vein clearing, vein flecking, stunting, slow decline, and quick decline) often were mistaken with other diseases and nutrient deficiency^8^. CTV, similar to another major citrus pathogen *‘Candidatus* Liberibacter spp.’ (causal agent of citrus greening/HLB), is graft transmissible and spread to other areas by propagation of infected buds. Therefore, dispersal of this pathogen could be reduced by use of healthy propagation material. Indexing of mother-plants using specific and reliable molecular detection techniques in the nursery would be an important step to prevent its spread^9^.

**Figure 1:**
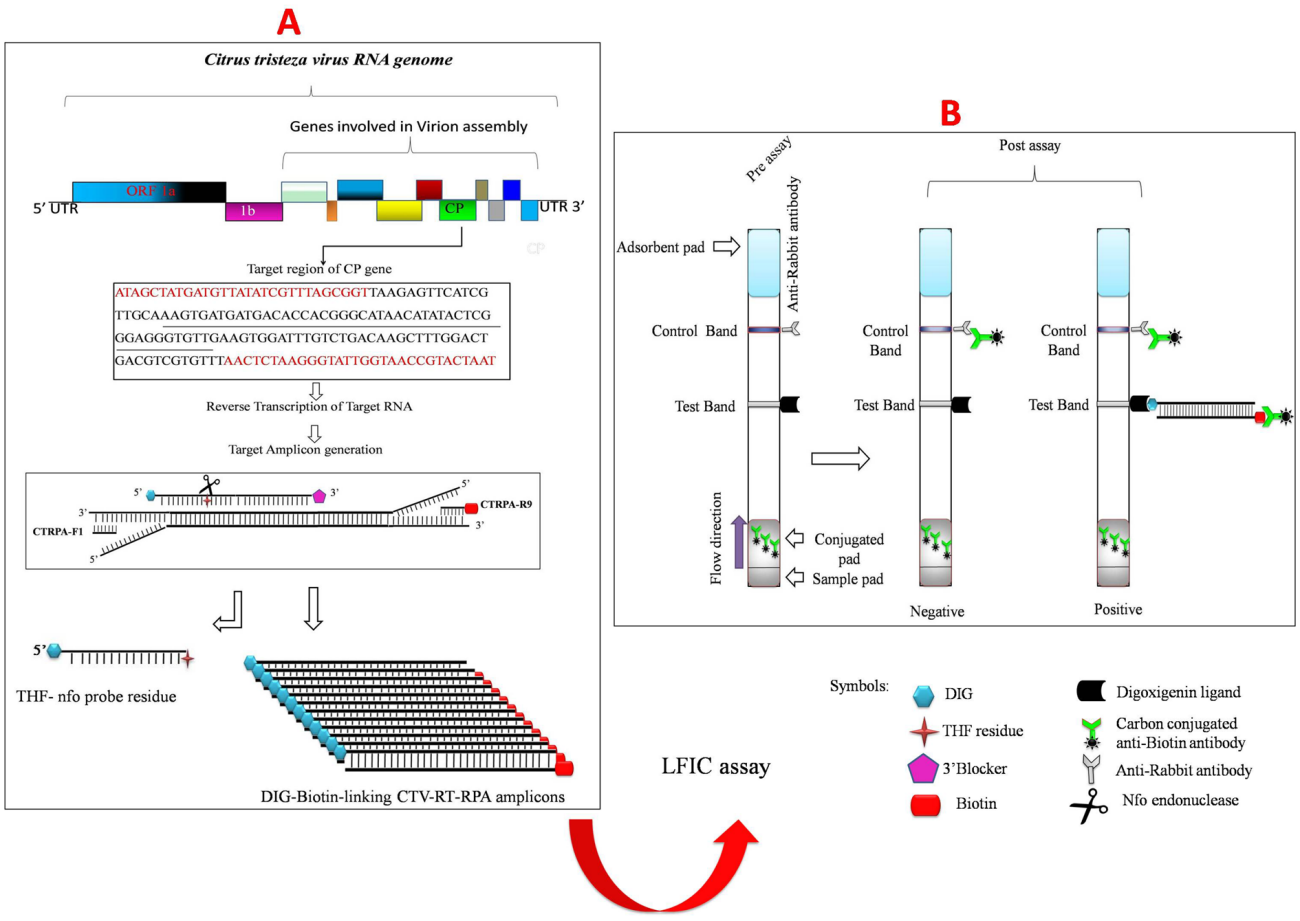
Schematic representation of the CTV-RT-RPA-LFICA principle for the detection of *Citrus tristeza virus* (**A**) Schematic representation of the CTV genome showing genomic open reading frames (ORFs) (top). Amplification of Digoxigenin-biotin-linking CTV coat protein gene (p25) target. The RT-RPA driven CTRPA-F1/CTRPA-R9-Btn primers first generate initial target templates required to anneal with the nfo probe at its complementary site. The nfo endonuclease enzyme cleaves the probe at the position of the THF residue and generates a new 3′OH site. Consequently, the 3′ end unblocked probe acts as primer for polymerization of the target region. The generated amplicons by processed probe co-join the digoxigenin and biotin in one DNA molecule. (**B**) End point detection of the digoxigenin-biotin linked RT-RPA product by lateral flow immunochromatographic sandwich assay. *UTR* untranslated region, *CP* coat protein gene, *DIG* digoxigenin, *THF* tetrahydrofuran.

In addition to biological indexing, many serological and molecular techniques have been employed for the detection of CTV such as, enzyme-linked immunosorbent assays (ELISA), dot immunobinding assays (DIBA), RT-PCR, RT-qPCR, electron microscopy, and Loop-mediated isothermal amplification (LAMP)^10–16^. These methods (excluding LAMP), have certain limitations inherently *i.e*. time consuming, labor intensive, require sophisticated expensive equipments, specific technical expertise and are not able to be used at point-of-care. Some disadvantages of LAMP include need for high temperature (65 °C), more amplification time, susceptible to carryover contaminations, and complex in primers design^17–20^. During development of any new detection method, concerns must account for sensitivity, specificity, simplicity, cost, robustness and rapidity^21^. The recombinase polymerase amplification (RPA) is a nucleic acid amplification technique that depends on the extension of primers induced by the recombination process and could be performed at isothermal temperature (37–42 °C) within 15–25 min^19,22^. RPA overcomes the limitations of many current methods as it does not need expensive instruments, provides rapid and reliable results under low-resource conditions and therefore one of the most promising new molecular diagnostic technologies^21^. The specific combination of enzymes involved in an isothermal RPA reaction are a recombinase, a strand displacing DNA polymerase and a single strand binding protein (SSB). The principle of the RPA technology mainly relies on the ATP dependent recombinase enzyme, which forms a nucleoprotein filament by binding the primers and scanning for the homologous double stranded DNA (dsDNA) sequence of the template to facilitate strand exchange at cognate sites. The SSB protein stabilizes the resulting D loop structure to perform the extension at the 3ˈ end of the invading primer using the complementary strand as a template by DNA polymerase I and accomplishes exponential amplification by cyclic repetition^23–27^. The amplified end product of the RPA reaction can be detected by different methods including agarose gel electrophoresis, colour-based visual detection, real-time fluorescence analysis with exo or fpg probes, and lateral flow immunochromatographic assays (LFICA). The LFICA-based detection system requires a Twist Amp nfo probe labeled with an antigenic molecule such as FAM/FITC/digoxigenin at the 5′ termini and labeled reverse primers with an antigenic biotin molecule at the 5′ termini. The amplified amplicons using these Twist Amp nfo probe and biotin labeled reverse primers can be detected on ‘sandwich immunochromatographic’ assays such as*,* the PCRD nucleic acid detector and Milenia GenLine HybriDetect strip (TwistDx Limited, Cambridge, UK)^27,28^. The different types of RPA-based assays have been reported to detect pathogens in animals^29–31^, humans^32,33^, and plants. Amongst the plant pathogens for which RPA detection assays have been developed are *Citrus yellow mosaic virus*^34^, *Ca*. L. asiaticus^27,35^, *Yam mild mosaic virus, Yam mosaic virus*^21^, *Little cherry virus*^36^, *plum pox virus*^37^, *Phytophthora* spp.^38^ and *begomoviruses*^22^. The aim of the present study was to develop a novel, rapid, robust and highly specific reverse transcription-RPA technique coupled with lateral flow immunochromatographic assay for CTV (working principle is illustrated in Fig. 1) and evaluate its efficacy in comparison with RT-PCR and RT-qPCR.

## Results

### Screening of CTV-RT-RPA-LFICA primers and specificity assessment

**Figure 2:**
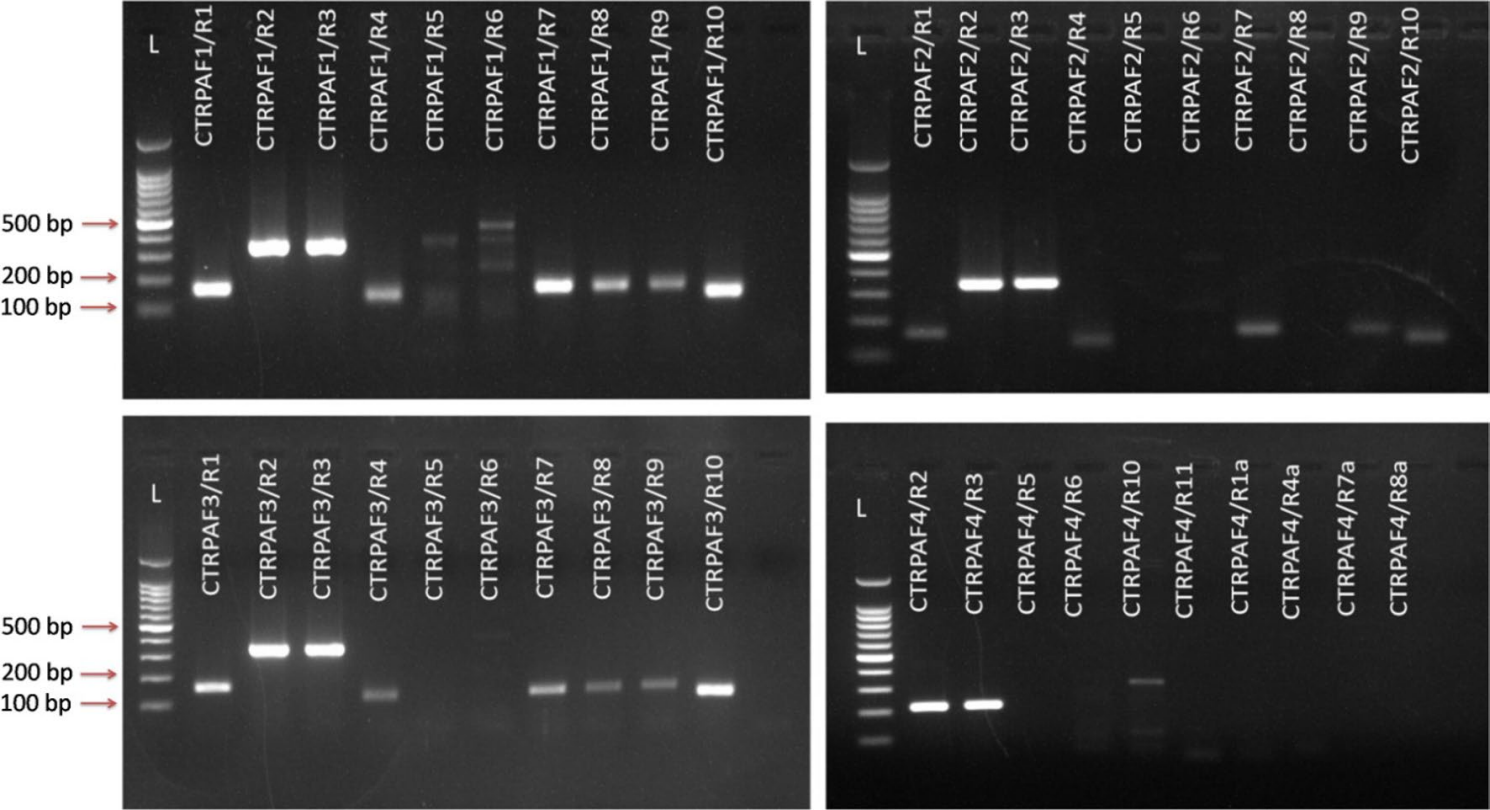
Primer optimization and screening. Several sets of primer combinations [4 forward (CTRPA-F1 to F4) and 15 reverse (CTRPA-R1 to R11)] were screened for selection of optimally working primer pairs. Lanes indicate the amplified product generated by different combinations of forward and reverse primers. Lanes L: 100 bp Marker.

**Figure 3:**
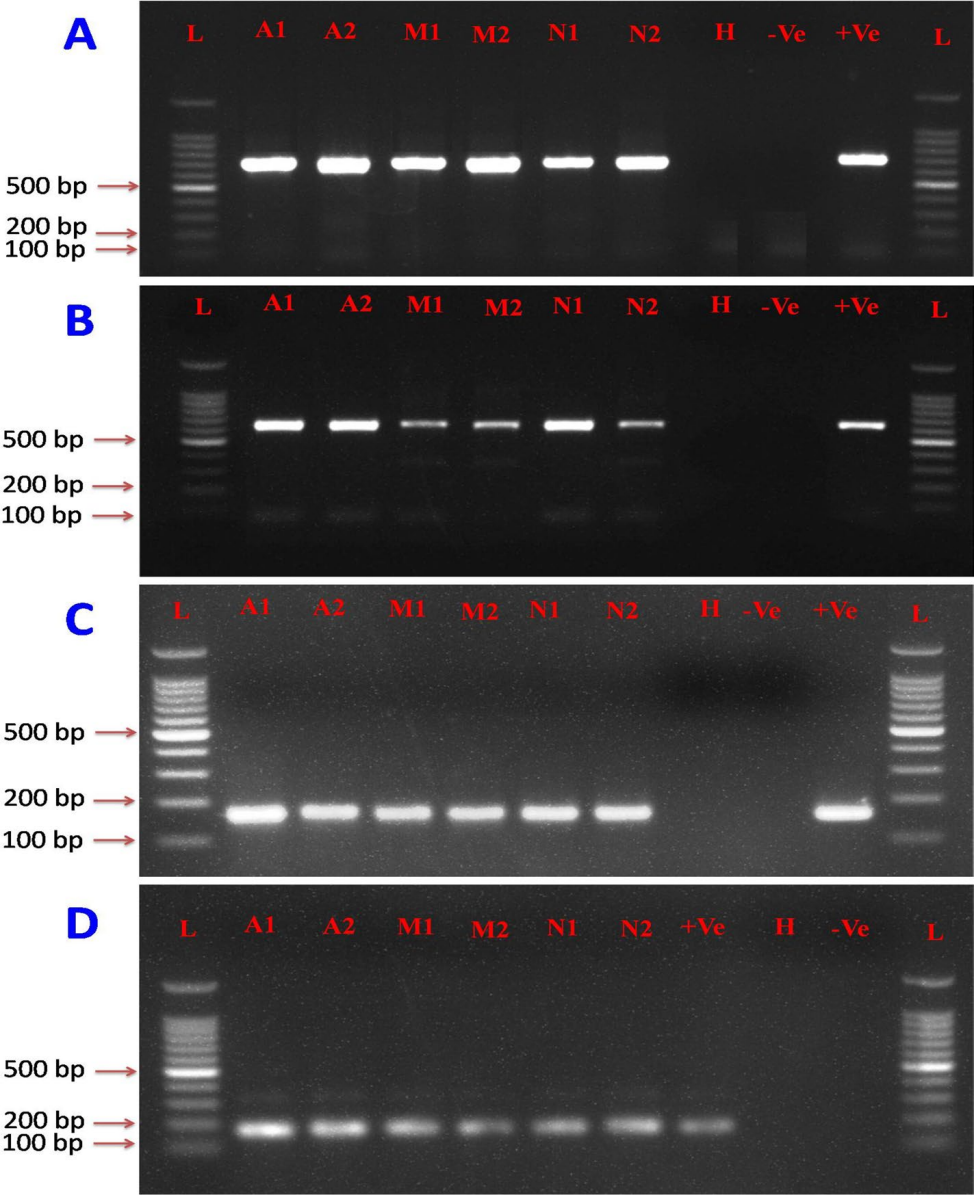
Agarose gel electrophoresis of RT-PCR and RT-RPA products of representative experimental samples; A1, A2, M1, M2, N1, N2, and H. (**A**) RT-PCR amplified amplicons using CN150/CN151, the coat protein gene specific primers of CTV, analyzed by 1% agarose gel electrophoresis. (**B**) RT-PCR amplified amplicons using p23-RBP-F/R, the RNA binding protein gene specific primers of CTV, were analyzed on 1% agarose gels. (**C**) RT-PCR amplified amplicons using CTRPA-F1/CTRPA-R9 coat protein gene specific primers of CTV analyzed by 1.5% agarose gels. (**D**) RT-RPA amplified amplicons with TwistAmp basic kit, using CTRPA-F1/CTRPA-R9 coat protein gene specific primers of CTV analyzed on 1.5% agarose gels.

Forty different combinations of forward and reverse primers (4 forward and 15 reverse) targeting a portion of the coat protein gene of CTV were analyzed for specificity and cross reactivity using different strains of CTV and other citrus pathogens with primer-BLAST software. During in silico analysis it was observed that all primer sets were unable to find complementary regions against other citrus infecting pathogen except CTV. Among these, 26 combinations of primers were identified as capable of amplifying target CTV RNA by conventional RT-PCR (Fig. 2). Optimally working primer sets from the initial RPA assays (CTRPAF1/R1, CTRPAF2/R1 and CTRPAF3/R1) were selected for RT-RPA-LFICA. The CTRPA-R9-Btn reverse primer was selected as it showed the minimum complementary energy (ΔG) − 4.41 kcal/mole between reverse primer and probe. The CTRPA-F1/R9-Btn combination was identified as the most optimally performing primer set which consistently amplified a ~ 165 bp specific region of the CTV- p25 gene and was therefore used for further optimization. The CTV positive control and the negative sample used in the present study were further confirmed using conventional RT-PCR. An expected size of ~ 672 bp amplicons with CN150/CN151 primers and ~ 630 bp with P23RBP-F/R primers was observed in CTV infected samples (A1, A2, A3, A4, M1, M2, M3, M4, N1, N2, N3 and N4) and no band was observed in the negative controls (HA1, HA2, HM1, HM2, HN1, HN2). However, amplicons of only six representative samples (A1, A2, M1, M2, N1 and N2) along with positive and negative controls were separated on 1% agarose gel (Fig. 3A,B). The CTV positive and negative samples were used to evaluate the specificity and efficacy of the RT-RPA primers (CTRPA-F1/CTRPA-R9) by conventional PCR. The expected amplification product of ~ 165 bp was observed in CTV positive samples whereas no amplification was observed in the healthy samples and non-template control (Fig. 3C). The PCR products amplified by RT-RPA primers were purified by gel elution and sequenced. In silico analyses confirmed sequences were specific for CTV.

### CTV-RT-RPA

Total RNAs isolated from CTV positive (A1, A2, A3, A4, M1, M2, M3, M4, N1, N2, N3 and N4) and negative citrus plants (HA1, HA2, HM1, HM2, HN1 and HN2) were reverse transcribed into single stranded cDNA. The standardization of the CTV-RT-RPA assay was carried out using cDNA as template. The best result was observed at 40 °C with 25 min reaction time. The end point detection of the RPA assay using agarose gel electrophoresis showed the expected ~ 165 band in CTV positive plants and no amplification was observed in control plants (Fig. 3D). The RPA product was gel eluted, sequenced and validated as CTV specific.

### Optimization of the CTV-RT-RPA-LFIC assay

**Figure 4:**
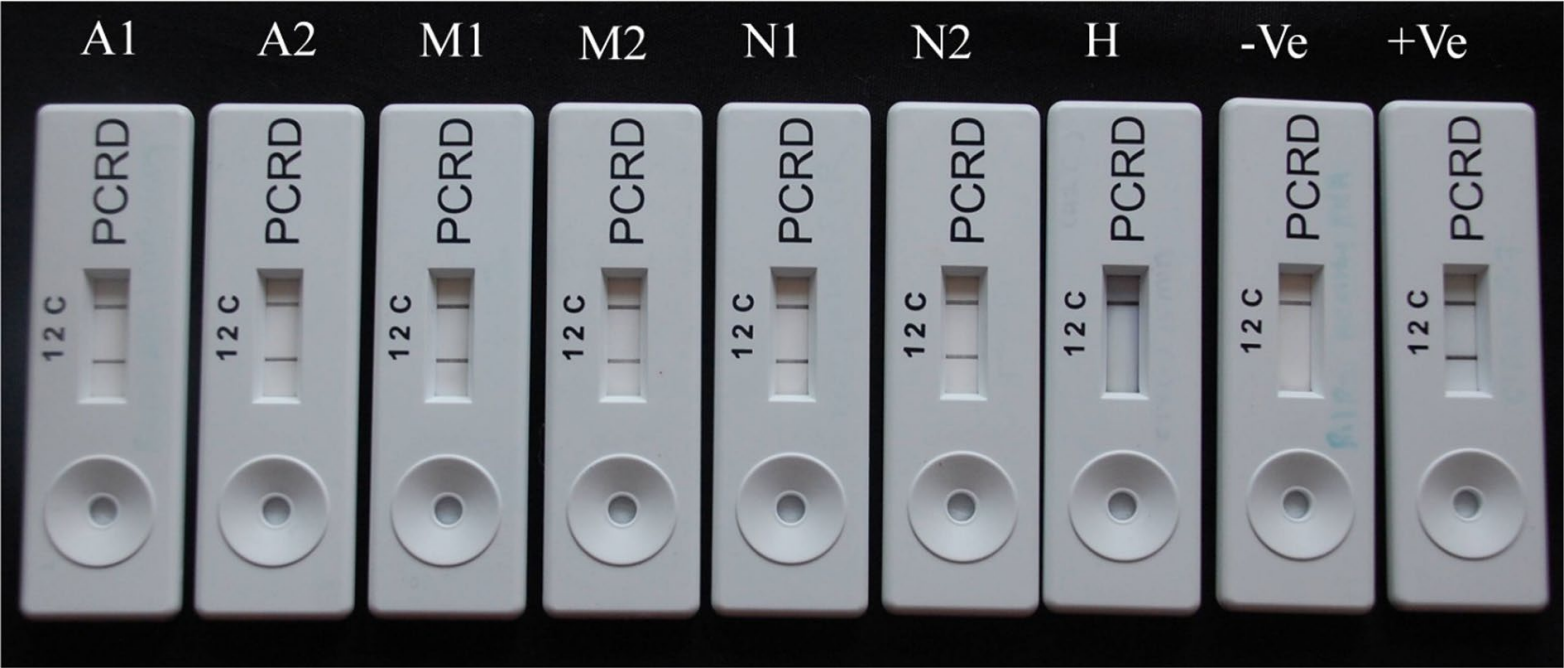
CTV-RT-RPA-LFICA using RNA as a template with three reaction lines: Line C is the control line; Line1 for detection of digoxigenin/biotin-labelled CTV amplicons; Line 2 is not used in the present study. A1, A2, M1, M2, N1, N2, and H represent experimental samples maintained in the screen house, H: Healthy control, −ve: negative control and +ve positive control.

**Figure 5:**
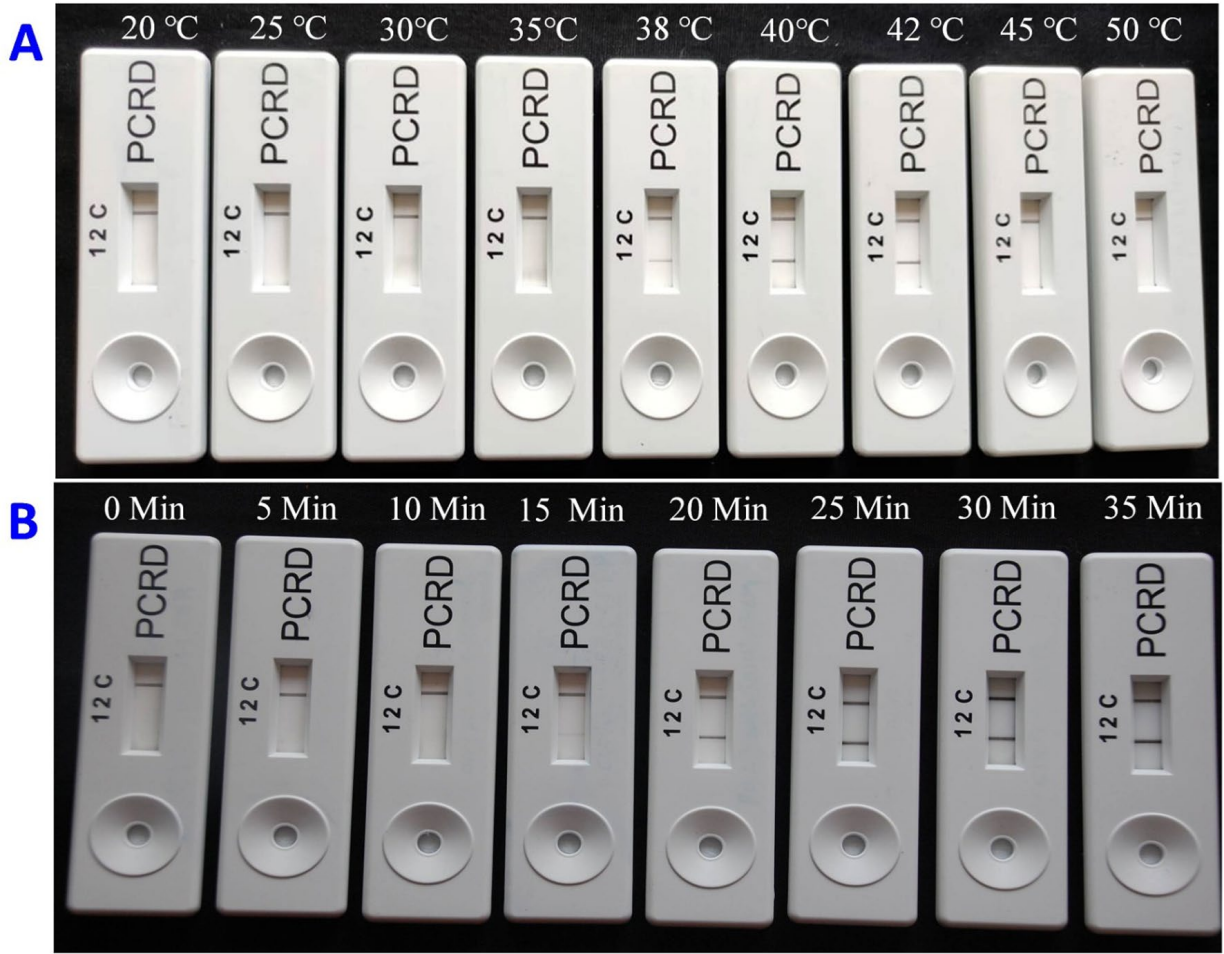
Determination of optimum reaction temperature and time. CTV-RT-RPA-LFICA was performed at different temperatures (**A**) and times (**B**) as represented in the figure. The assay works effectively in a temperature range of 37–42 °C with best visibility of the test line (T-line) at 40–42 °C. The assay starts developing a test line after 15 min of incubation time with a very faint line and optimal visibility of the test line (T-line) in the ranges 20, 25, 30 and 35 min.

The CTV-RT-RPA-LFIC assay was optimized using synthesized cDNA and the total RNA isolated from CTV positive citrus plants. A PCRD nucleic acid detector was used to capture and detect CTV specific amplified double labeled products generated by RT-RPA. Upon application of the amplified products on the sample port, the carbon conjugated anti-biotin antibodies react with the Dig/biotin labeled amplicons. The complex of carbon conjugated antibody-Dig/biotin amplicons get captured at the test line (T-lines) and control line (C-line) by manifestation of a coloured visible line. The appearance of both lines occurring simultaneously within 2 min after application of the amplified product was considered as a positive result for CTV whereas development of only the control line indicated negative results (Fig. 4). It was observed that the sample with higher CTV titer required minimum time (within 60 s) to develop a more intense-visible line (T-lines). It was also observed that RNA templates required more incubation time (20–25 min) than cDNA as initial template. The optimal results of the CTV-RT-RPA-LFIC assay were observed at 40–42 °C (Fig. 5A) at a reaction incubation time of 20–25 min from RNA as initial template and 15 min with cDNA as template (Fig. 5B). The validation of RT-RPA-LFICA performed with an *in planta* culture correctly identified all examined samples consistently with RT-PCR and RT-qPCR. All assays showed similar performance with known CTV positive and healthy samples (Figs. 3A,B, 4, 9).

### Sensitivity and specificity evaluation of the CTV-RT-RPA-LFIC assay

**Figure 6:**
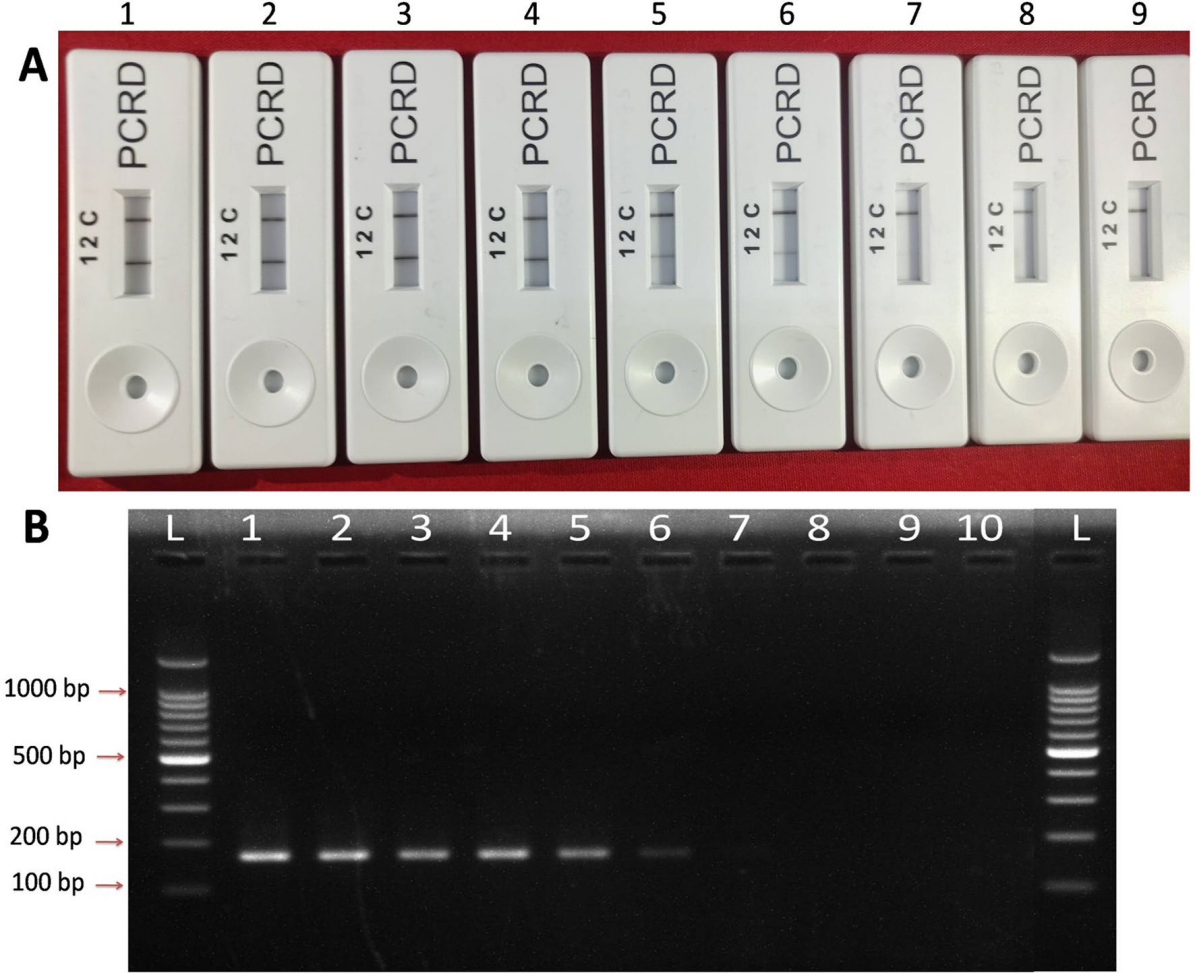
Detection limit analysis of CTV-RT-RPA-LFICA versus conventional RT-PCR with primer CTRPAF1/R9 for CTV using tenfold serially diluted cDNA as template synthesized from in vitro-transcribed RNA transcripts. (**A**) The amplified RT-RPA products were analyzed by the PCRD nucleic acid detector. The RNA concentration used for cDNA synthesis was, Lane-1 = 141 ng (RNA copies = 3.773 × 10^11^), Lane-2 = 14.1 ng (RNA copies = 3.773 × 10^10^), Lane-3 = 1.41 ng (RNA copies = 3.773 × 10^9^), Lane-4 = 0.141 ng (RNA copies = 3.773 × 10^8^), Lane-5 = 0.0141 ng (RNA copies = 3.773 × 10^7^), Lane-6 = 0.00141 ng (RNA copies = 3.773 × 10^6^), Lane-7 = 0.000141 ng (RNA copies = 3.773 × 10^5^) and Lane-8 = 0.0000141 ng (RNA copies = 3.773 × 10^4^). Lane 9: NTC (Non-template control). (**B**) Electrophoretic migration in a 1.5% agarose gel of the amplification product obtained from tenfold cDNA serial dilution**.** Lane L, 100 bp Ladder; the RNA concentration used for cDNA synthesis was, Lane-1 = 141 ng (RNA copies = 3.773 × 10^11^), Lane-2 = 14.1 ng (RNA copies = 3.773 × 10^10^), Lane-3 = 1.41 ng (RNA copies = 3.773 × 10^9^), Lane-4 = 0.141 ng (RNA copies = 3.773 × 10^8^), Lane-5 = 0.0141 ng (RNA copies = 3.773 × 10^7^), Lane-6 = 0.00141 ng (RNA copies = 3.773 × 10^6^), Lane-7 = 0.000141 ng (RNA copies = 3.773 × 10^5^), Lane-8 = 0.0000141 ng (RNA copies = 3.773 × 10^4^) and Lane-9 = 0.00000141 ng (RNA copies = 3.773 × 10^3^). Lane-10: NTC.

**Figure 7:**
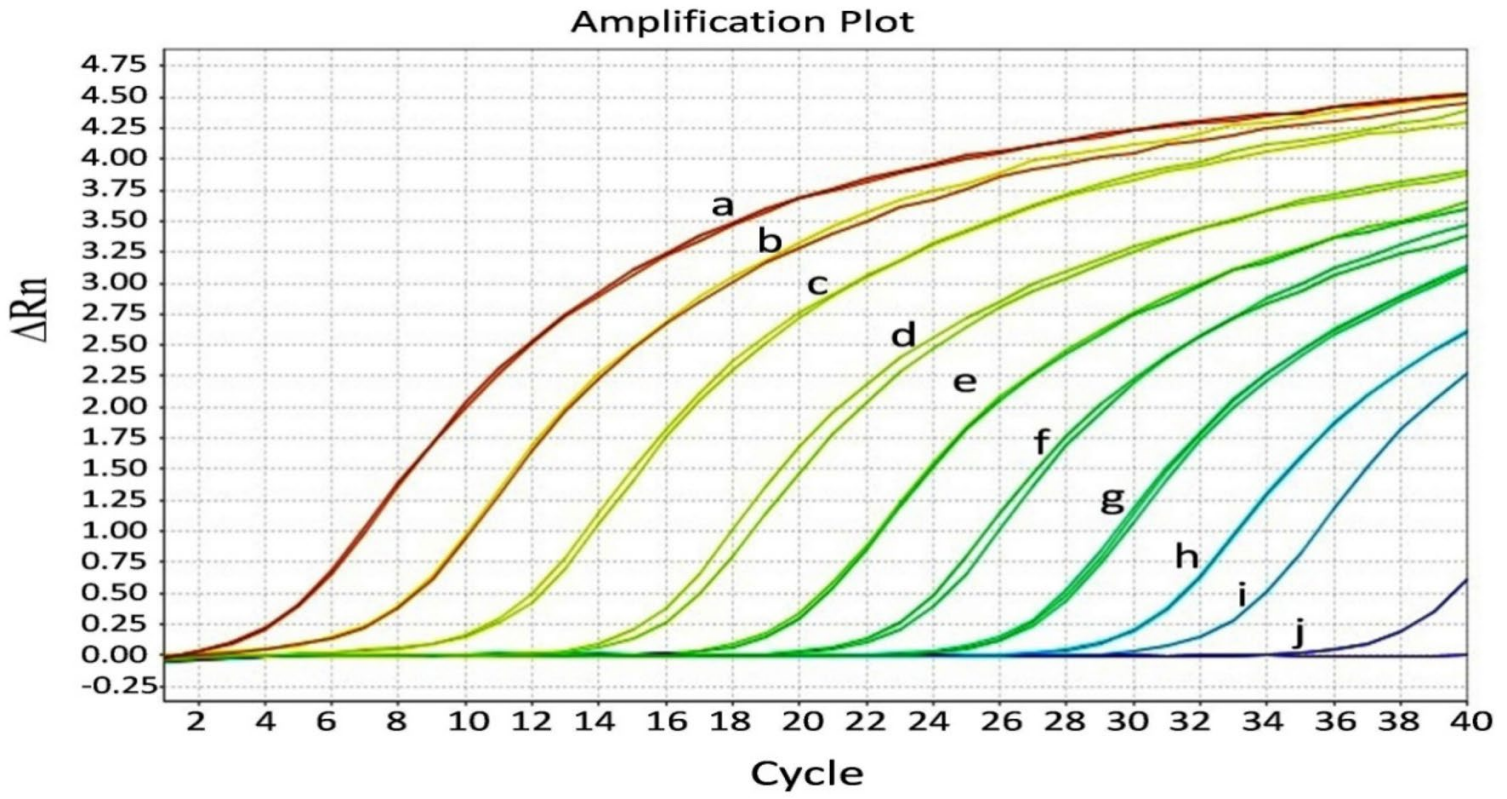
Detection limit analysis for TaqMan reverse transcription-qPCR assay with P25F/R-CTV FAM primer pair probe using tenfold serially diluted cDNA as a template synthesized from in vitro-transcribed RNA transcripts. Amplification plot generated using tenfold serial dilution of cDNA to cross verify the sensitivity of TaqMan-qPCR with CTV-RT-RPA-LFICA. The RNA concentration used for cDNA synthesis was, Line-a = 141 ng (RNA copies = 3.773 × 10^11^), Line-b = 14.1 ng (RNA copies = 3.773 × 10^10^), Line-c = 1.41 ng (RNA copies = 3.773 × 10^9^), Line-d = 0.141 ng (RNA copies = 3.773 × 10^8^), Line-e = 0.0141 ng (RNA copies = 3.773 × 10^7^), Line-f = 0.00141 ng (RNA copies = 3.773 × 10^6^), Line-g = 0.000141 ng (RNA copies = 3.773 × 10^5^), Line-h = 0.0000141 ng (RNA copies = 3.773 × 10^4^), Line-i = 0.00000141 ng (RNA copies = 3.773 × 10^3^), and Line-j = 0.000000141 ng (RNA copies = 3.773 × 10^2^). A cycle threshold (Ct) value of 4.78 was obtained for the 141 ng (Line-a) in vitro RNA transcripts. Whereas very little or no fluorescence was observed for 0.000000141 ng (Line-j).

**Figure 8:**
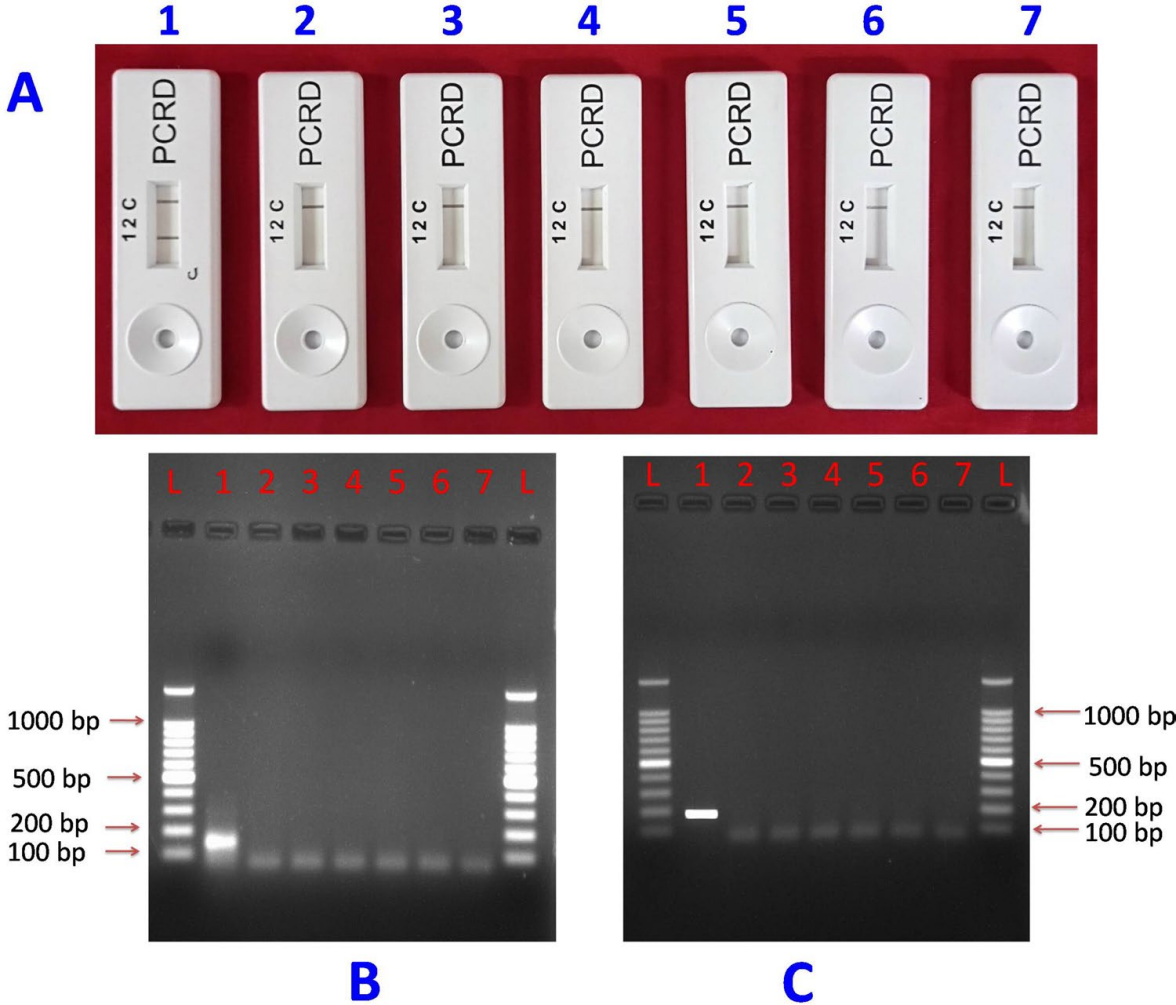
Specificity analysis of CTV-RT-RPA-LFICA using primer CTRPAF1/R9 with other major citrus pathogen. (**A**) The amplified RT-RPA products were analyzed by the PCRD nucleic acid detector. (**B**) Basic RT-RPA amplified products resolved on 1.5% agarose gel electrophoresis. (**C**) RT-PCR amplicon of CTV using primer CTRPAF1/R9. Lane L, 100 bp DNA Ladder; Lane 1, amplified products of CTV; Lane 2 to 5, reaction products of citrus yellow mosaic virus, Indian citrus ringspot virus, *Ca*. L. asiaticus, and Phytoplasma, respectively; Lane 6, healthy plant control and Lane 7, reaction control.

RT-RPA coupled with a sandwich immunochromatographic assay consistently detected target cDNA of CTV up to 10^–5^ serial dilution synthesized from 141 ng of in vitro-transcribed RNA as the initial template. A faint band was observed for the 10^–6^ serial dilution of target cDNA (corresponding to 3.77 × 10^5^ RNA copies). The test (T-line) band intensity approximately correlated with initial template concentration. The detection limit of the CTV-RT-RPA-LFIC assay was ≤ 141 fg of RNA when converted into cDNA and used as a template (3.77 × 10^5^ RNA copies) (Fig. 6A). However, the assay detected ≤ 0.23 ng/µl (6.288 × 10^8^ RNA copies) when RNA was used directly as template. The detection limit for conventional RT-PCR was nearly 10^–5^ serial dilution (3.77 × 10^6^ RNA copies) of target cDNA (Fig. 6B) and the detection limit of TaqMan real-time PCR assay was recorded near > 10^–8^ serial dilution of target cDNA with Ct value > 33.3 (3.773 × 10^3^ RNA copies) (Fig. 7). The primer pair was modified such that they contained unique nucleotide stretches and did not mix with the template before adding enzyme to avoid any non-specific binding of recombinase enzyme during the reaction. Specificity analysis showed that the developed CTV-RT-RPA-LFIC assay was highly specific to CTV and failed to detect any other non target citrus pathogens (Fig. 8).

### Validation of the CTV-RT-RPA-LFIC assay using field samples

**Figure 9:**
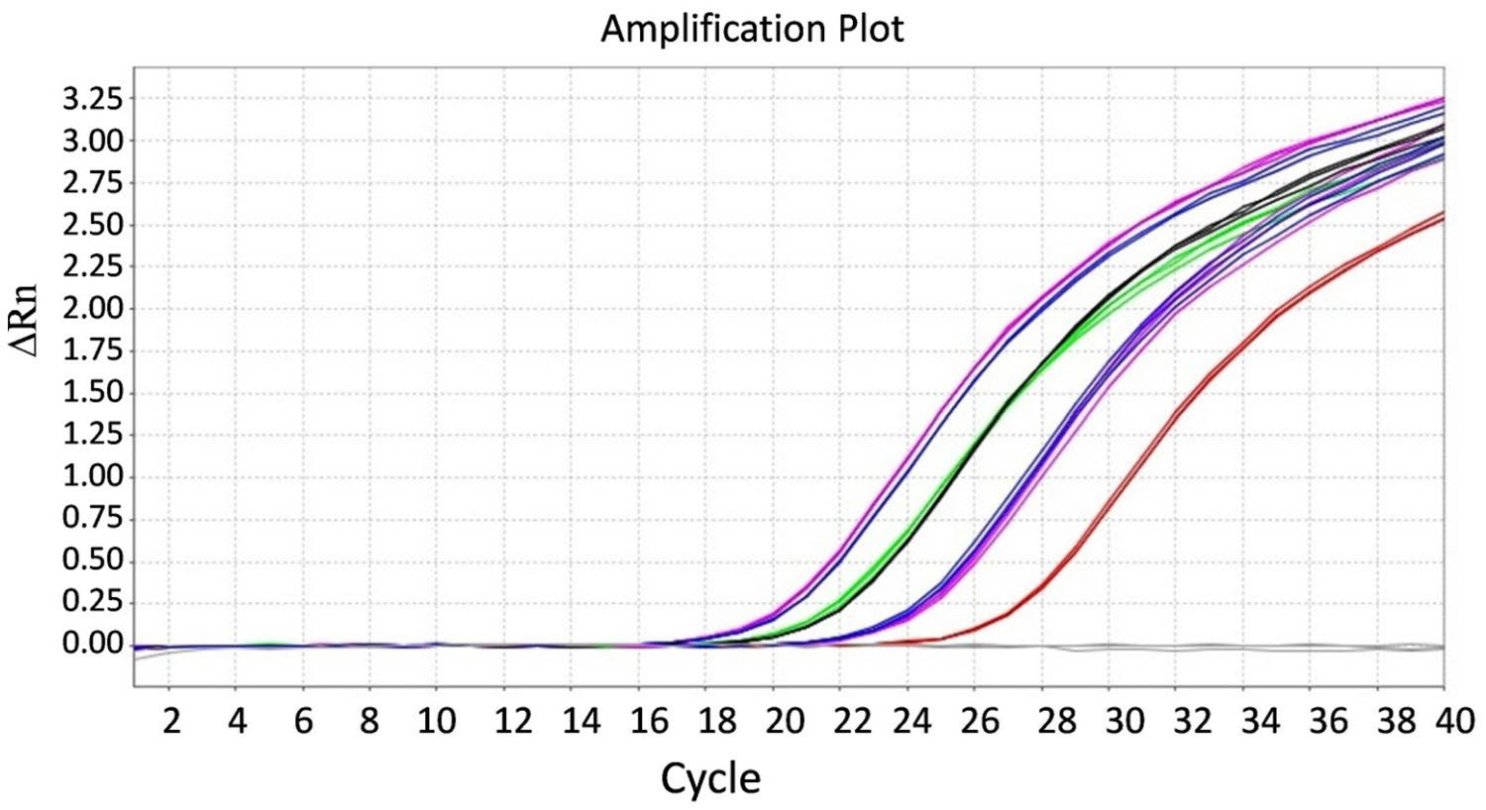
Validation of the CTV-RT-RPA-LFIC assay using TaqMan RT-qPCR with P25F/R-CTV FAM primer pair probe using cDNA as template. Amplification plot for representative experimental samples; A1, A2, M1, M2, N1, N2, and positive control showing average cycle threshold (Ct) value, 18.76, 17.34, 21.38, 17.50, 24.34, and 19.2 and 21.51 respectively. No fluorescence signal was observed with NTC (Non-template control) and H: Healthy control.

Results obtained with CTV-RT-RPA-LFIC assays were confirmed by TaqMan based real-time PCR (Fig. 9) and conventional RT-PCR (Fig. 3A,B). To validate and evaluate the feasibility of the CTV-RT-RPA-LFIC assay, 80 different samples (including 6 healthy samples) suspected as CTV were tested and compared with TaqMan RT-qPCR and RT-PCR. The results showed that 43 samples were identified as positive for CTV by RT-RPA-LFICA (Supplementary Fig. S1), while 46 samples were validated as positive by RT-qPCR (Ct value ranging from 18.45 to 33.9), and the conventional RT-PCR based on primer pair CN150/151 detected 41 samples as positive. However, conventional RT-PCR based on primer pair CTRPAF1/R9 detected 42 samples as CTV positive. CTV positive rate was 53.75% (43/80), 57.5% (46/80) and 51.25% (41/80) with RT-RPA-LFICA, RT-qPCR and RT-PCR respectively. Out of 80 samples, CTV-RT-RPA-LFICA and the conventional RT-PCR failed to detect CTV only in three samples (L6P1, KP-5, SGL-2) which were confirmed positive by the TaqMan RT-qPCR (Ct value > 33.5). The concordance rate of the CTV-RT-RPA-LFICA with the TaqMan RT-qPCR assay was 96.25% (77/80) (Table 1) with the kappa value of 0.926. These results indicate that the developed assay demonstrated an excellent diagnostic agreement with RT-qPCR and would be effective for the detection of CTV in field samples.

**Table 1:** Specificity analysis of CTV-RT-RPA-LFICA compared with RT-PCR, and TaqMan RT-qPCR for the detection of *Citrus tristeza virus*.

Sr. No	Sample code	Cultivar	Location	Checked for *“*CTV”			
				CTV-RT-RPA-LFICA	RT-PCR with RT-RPA primers (CTRPA-F1/R9)	RT-PCR with p25 (coat protein gene) based primers (CN150/CN151)	TaqMan RT-qPCR
1	BT R1P3	Nagpur mandarin (*Citrus reticulata)*	Chhindwara, Madhya Pradesh	−	−	−	−
2	BT R2P7	Nagpur mandarin (*Citrus reticulata)*	Chhindwara, Madhya Pradesh	−	−	−	−
3	BT R3P13	Nagpur mandarin (*Citrus reticulata)*	Chhindwara, Madhya Pradesh	−	−	−	−
4	BT R4P16	Nagpur mandarin (*Citrus reticulata)*	Chhindwara, Madhya Pradesh	+	+	+	+
5	BT R4P7	Nagpur mandarin (*Citrus reticulata)*	Chhindwara, Madhya Pradesh	−	−	−	−
6	BT R5P2	Nagpur mandarin (*Citrus reticulata)*	Chhindwara, Madhya Pradesh	+	+	+	+
7	BT R6P1	Nagpur mandarin (*Citrus reticulata)*	Chhindwara, Madhya Pradesh	−	−	−	−
8	BT R6P10	Nagpur mandarin (*Citrus reticulata)*	Chhindwara, Madhya Pradesh	+	+	+	+
9	BT R8P14	Nagpur mandarin (*Citrus reticulata)*	Chhindwara, Madhya Pradesh	+	+	+	+
10	BT R7P17	Nagpur mandarin (*Citrus reticulata)*	Chhindwara, Madhya Pradesh	−	−	−	−
11	BT2 R2P7	Nagpur mandarin (*Citrus reticulata)*	Chhindwara, Madhya Pradesh	−	−	−	−
12	BT2 R1P12	Nagpur mandarin (*Citrus reticulata)*	Chhindwara, Madhya Pradesh	−	−	−	−
13	BT2 R4P9	Nagpur mandarin (*Citrus reticulata)*	Chhindwara, Madhya Pradesh	−	−	−	−
14	BT2 R6P21	Nagpur mandarin (*Citrus reticulata)*	Chhindwara, Madhya Pradesh	+	+	+	+
15	BT2 R15P1	Nagpur mandarin (*Citrus reticulata)*	Chhindwara, Madhya Pradesh	+	+	+	+
16	BT2 R12P2	Nagpur mandarin (*Citrus reticulata)*	Chhindwara, Madhya Pradesh	+	+	−	+
17	L1P1	Sweet orange (*Citrus sinensis*)	ICAR-CCRI, Nagpur, Maharashtra	+	+	+	+
18	L1P2	Sweet orange (*Citrus sinensis*)	ICAR-CCRI, Nagpur, Maharashtra	+	+	+	+
19	L1P3	Sweet orange (*Citrus sinensis*)	ICAR-CCRI, Nagpur, Maharashtra	+	+	+	+
20	L2P2	Sweet orange (*Citrus sinensis*)	ICAR-CCRI, Nagpur, Maharashtra	+	+	+	+
21	L2P3	Sweet orange (*Citrus sinensis*)	ICAR-CCRI, Nagpur, Maharashtra	+	+	+	+
22	L2P5	Sweet orange (*Citrus sinensis*)	ICAR-CCRI, Nagpur, Maharashtra	+	+	+	+
23	L3P3	Sweet orange (*Citrus sinensis*)	ICAR-CCRI, Nagpur, Maharashtra	+	+	+	+
24	L3P5	Sweet orange (*Citrus sinensis*)	ICAR-CCRI, Nagpur, Maharashtra	+	+	+	+
25	L4P4	Sweet orange (*Citrus sinensis*)	ICAR-CCRI, Nagpur, Maharashtra	+	+	+	+
26	L4P5	Sweet orange (*Citrus sinensis*)	ICAR-CCRI, Nagpur, Maharashtra	+	+	+	+
27	L5P3	Sweet orange (*Citrus sinensis*)	ICAR-CCRI, Nagpur, Maharashtra	+	+	+	+
28	L5P6	Sweet orange (*Citrus sinensis*)	ICAR-CCRI, Nagpur, Maharashtra	+	+	+	+
29	L5P7	Sweet orange (*Citrus sinensis*)	ICAR-CCRI, Nagpur, Maharashtra	+	+	+	+
30	L6P1	Sweet orange (*Citrus sinensis*)	ICAR-CCRI, Nagpur, Maharashtra	−	−	−	+
31	L7P3	Sweet orange (*Citrus sinensis*)	ICAR-CCRI, Nagpur, Maharashtra	+	+	+	+
32	L7P7	Sweet orange (*Citrus sinensis*)	ICAR-CCRI, Nagpur, Maharashtra	+	+	+	+
33	L8P5	Sweet orange (*Citrus sinensis*)	ICAR-CCRI, Nagpur, Maharashtra	+	+	+	+
34	L8P7	Sweet orange (*Citrus sinensis*)	ICAR-CCRI, Nagpur, Maharashtra	+	+	+	+
35	L9P1	Sweet orange (*Citrus sinensis*)	ICAR-CCRI, Nagpur, Maharashtra	+	+	+	+
36	L9P6	Sweet orange (*Citrus sinensis*)	ICAR-CCRI, Nagpur, Maharashtra	−	−	−	−
37	L9P7	Sweet orange (*Citrus sinensis*)	ICAR-CCRI, Nagpur, Maharashtra	+	+	+	+
38	L9P11	Sweet orange (*Citrus sinensis*)	ICAR-CCRI, Nagpur, Maharashtra	+	+	+	+
39	L3P2	Sweet orange (*Citrus sinensis*)	ICAR-CCRI, Nagpur, Maharashtra	+	−	−	+
40	L3P4	Sweet orange (*Citrus sinensis*)	ICAR-CCRI, Nagpur, Maharashtra	+	+	+	+
41	L5P9	Sweet orange (*Citrus sinensis*)	ICAR-CCRI, Nagpur, Maharashtra	+	+	+	+
42	L2P11	Sweet orange (*Citrus sinensis*)	ICAR-CCRI, Nagpur, Maharashtra	+	+	+	+
43	L4P6	Sweet orange (*Citrus sinensis*)	ICAR-CCRI, Nagpur, Maharashtra	+	+	+	+
44	L2P7	Sweet orange (*Citrus sinensis*)	ICAR-CCRI, Nagpur, Maharashtra	+	+	+	+
45	L4P7	Sweet orange (*Citrus sinensis*)	ICAR-CCRI, Nagpur, Maharashtra	+	+	+	+
46	L4P8	Sweet orange (*Citrus sinensis*)	ICAR-CCRI, Nagpur, Maharashtra	+	+	+	+
47	L4P11	Sweet orange (*Citrus sinensis*)	ICAR-CCRI, Nagpur, Maharashtra	+	+	+	+
48	Nm-2ac-1	Nagpur mandarin (*Citrus reticulata)*	Wardha, Maharashtra	−	−	−	−
49	Nm-2ac-2	Nagpur mandarin (*Citrus reticulata)*	Wardha, Maharashtra	−	−	−	−
50	Nm-2ac-3	Nagpur mandarin (*Citrus reticulata)*	Wardha, Maharashtra	−	−	−	−
51	Nm-2ac-4	Nagpur mandarin (*Citrus reticulata)*	Wardha, Maharashtra	−	−	−	−
52	Nm-5 a-1	Nagpur mandarin (*Citrus reticulata)*	Wardha, Maharashtra	−	−	−	−
53	Nm-5 a -2	Nagpur mandarin (*Citrus reticulata)*	Wardha, Maharashtra	−	−	−	−
54	Nm-5 a -3	Nagpur mandarin (*Citrus reticulata)*	Wardha, Maharashtra	−	−	−	−
55	Nm-5 a-4	Nagpur mandarin (*Citrus reticulata)*	Wardha, Maharashtra	−	−	−	−
56	YNS1	Sweet orange (*Citrus sinensis*)	Baramati, Pune Maharashtra	+	+	+	+
57	YNS1	Sweet orange (*Citrus sinensis*)	Baramati, Pune, Maharashtra	+	+	+	+
58	YNS1	Sweet orange (*Citrus sinensis*)	Baramati, Pune, Maharashtra	+	+	+	+
59	YNS1	Sweet orange (*Citrus sinensis*)	Baramati, Pune, Maharashtra	+	+	+	+
60	KP-2	Acid lime (*Citrus aurantifolia*)	Government Nursery, Ajara, Kolhapur, Maharashtra	−	−	−	−
61	KP-3	Acid lime (*Citrus aurantifolia*)	Government Nursery, Chandgad, Kolhapur, Maharashtra	−	−	−	−
62	KP-4	Acid lime (*Citrus aurantifolia*)	Government Nursery, Jaysingpur, Kolhapur, Maharashtra	−	−	−	−
63	KP-5	Acid lime (*Citrus aurantifolia*)	Agriculture College, Kolhapur, Maharashtra	−	−	−	+
64	KP-6	Acid lime (*Citrus aurantifolia*)	Bharat Nursery, Varnul, Kolhapur, Maharashtra	−	−	−	−
65	SGL-1	Acid lime (*Citrus aurantifolia*)	Government Nursery Kupwad, Sangli, Maharashtra	−	−	−	−
66	SGL-2	Acid lime (*Citrus aurantifolia*)	Agriculture research center, Kasbe Digraj, Sangli, Maharashtra	−	−	−	+
67	NGR2P2	Khasi mandarin (*Citrus reticulata*)	Nagaland	−	−	−	−
68	NGR3P1	Khasi mandarin (*Citrus reticulata*)	Nagaland	−	−	−	−
69	NGR6P1	Khasi mandarin (*Citrus reticulata*)	Nagaland	+	+	+	+
70	NGR7P1	Khasi mandarin (*Citrus reticulata*)	Nagaland	−	−	−	−
71	NGR2P1	Kinnow mandarin (*Citrus reticulata*)	Nagaland	−	−	−	−
72	NGR5P13	Sikkim mandarin Orange (*Citrus reticulata*)	Nagaland	+	+	+	+
73	NGR5P14	Sikkim mandarin Orange (*Citrus reticulata*)	Nagaland	*−*	−	−	−
74	NGR6P16	Sikkim mandarin Orange (*Citrus reticulata*)	Nagaland	+	+	+	+
75	HA1 (Healthy)	Acid lime (*Citrus aurantifolia*)	ICAR-CCRI, Nagpur, Maharashtra	−	−	−	−
76	HA2 (Healthy)	Acid lime (*Citrus aurantifolia*)	ICAR-CCRI, Nagpur, Maharashtra	−	−	−	−
77	HM1(Health)	Sweet orange (*Citrus sinensis*)	ICAR-CCRI, Nagpur, Maharashtra	−	−	−	−
78	HM2 (Healthy)	Sweet orange (*Citrus sinensis*)	ICAR-CCRI, Nagpur, Maharashtra	−	−	−	−
79	HN1 (Healthy)	Nagpur mandarin (*Citrus reticulata)*	ICAR-CCRI, Nagpur, Maharashtra	−	−	−	−
80	HN2 (Healthy)	Nagpur mandarin (*Citrus reticulata)*	ICAR-CCRI, Nagpur, Maharashtra	−	−	−	−

## Discussion

CTV is one of the most economically important pathogens of citrus and has destroyed millions of citrus trees worldwide^1^. The virus infection causes reduction in yield and quality of citrus fruits and induced stem pitting and devastating quick-decline symptoms. Citrus is mainly a vegetatively propagated crop and the major pathogens are transmitted through disease-infected buds and propagating planting material. In the field, the horizontal transmission is by aphid vectors in a semi-persistent manner. To prevent outbreaks of the disease, indexing of planting material is a crucial step that requires a specific and reliable virus detection techniques^9^. Numerous techniques have been developed for CTV detection but most of them have limitations viz., time consuming, requirement of expensive equipment and trained personnel. RT-PCR and RT-qPCR are the most acceptable techniques for CTV detection but require well setup laboratory, equipment and personel. Therefore, there is need to develop a rapid, robust and reliable on-site detection technique to assist in the certification of virus-free planting material. The RPA approach is an emerging isothermal, low cost, rapid, and point-of-care diagnostic tool^23^. It is a highly sensitive, reliable nucleic acid based method and has become a rapid detection tool for many pathogens including viruses^34^, bacteria^19,27^, and *Phytophthora* species^39^. It is also used for detection of RNA viruses without the need for a separate step to synthesize cDNA by RT-RPA^21,36,37,40^. This technology has the potential to be a promising alternative to RT-qPCR^41^. Enzymes (recombinase, SSB and strand displacement DNA polymerase) are required for exponential amplification of the target template^23^. To target the RNA template, extra reverse transcriptase enzyme need to be added to the reaction. Present study is first to report the development of a robust reverse transcription recombinase polymerase-based isothermal amplification technique coupled with lateral flow immunochromatographic assay (CTV-RT-RPA-LFICA) for the rapid detection of CTV.

The specificity of the optimized RT-RPA primers selected to amplify the CTV specific coat protein gene (CTV-p25) demonstrated by comparison with conventional RT-PCR and sequencing of PCR products as cognate gene specific. The analysis of the products of basic RPA by agarose electrophoresis requires an extra chloroform/isoamyl alcohol purification step to reduce the crowding and complexity of the amplified RPA product on the agarose gel for better band visibility compared to non-purified RT-RPA products (Fig. 3D). The specificity of the RT-RPA primers were also judged by in silico analysis (primer BLAST) that specifically detected the target pathogen and showed no cross reactivity with other major citrus pathogens. Hetero-dimer analysis using the Oligo Analyzer tool was performed between probe and reverse primers to obtain the realistic results on lateral flow immunochromatographic assay. Efforts were also made to obtain a minimum ∆G (free energy of the oligo sequence binding to its complement site) between probe and reverse primer. The ∆G value of − 4.41 kcal/mole for probe and CTRPA-R9 was observed as best to achieve reliable results owing to less complementarity between reverse primer and probe.

CTV-RT-RPA-LFICA developed in the present investigation was rapid, needed 20–25 min for amplification and 5 min for visualization. Application of RT-RPA amplified products on the sample port, the test line (T-lines) and control line (C-line) were visible as coloured bands with CTV positive samples simultaneously within 2 min, whereas development of color with only the C-line indicated negative results (Fig. 4). The assay needs less RT-RPA product (0.5 µl) for end point detection, the visualization process on the PCRD detector, as compared with other techniques. The results also suggested that RNA as an initial template needed more reaction incubation time (20–25 min) compared to cDNA as template (15 min). Higher pathogen titer in the sample would require much less time (~ 60 s) to develop more intense color band in T-lines. The reaction works at low isothermal temperature of 40 °C in a simple dry bath without expensive thermal cyclers (Fig. 5A). The reaction also could be performed within a broad range of temperatures (37–42 °C)^42^. The reaction needed a single set of primers, whereas LAMP needs as many as four to six primers^39^. The quick lateral flow immunochromatographic assay optimized using the Twist Amp nfo probe which saved an additional 60 to 80 min compared to RPA where products have to be visualized by agarose gel electrophoresis.

The sensitivity of RT-RPA-LFICA was shown to be equal or better compared to conventional RT-PCR, but less sensitive compared to the TaqMan-RT-qPCR (Figs. 6, 7 and Supplementary Fig. S1). The ability of RT-RPA-LFICA to detect the CTV using genomic RNA as template with a limit up to 0.23 ng/µl of RNA was demonstrably a strong advantage compared to RT-PCR and TaqMan-qPCR. Specificity analysis showed that the developed CTV-RT-RPA-LFIC assay is highly specific to CTV and did not cross react with any other non-target citrus pathogens (Fig. 8). The specificity of the assay was further validated by testing seventy-four field grown CTV-suspected samples and compared with RT-PCR and RT-qPCR. These results confirm the robustness and specificity of the newly-designed RT-RPA-LFICA primers-probes and demonstrated excellent diagnostic agreement with RT-qPCR (kappa value = 0.926). The major advantage of this technique is that the result can be easily judged as positive or negative visually and thus a great potential to be used as a point-of-care diagnostic tool and a valuable tool for nursery citrus bud wood certification programs.

## Materials and methods

### Maintenance of CTV infected plants, sample collection and processing

CTV infected (sample code: A1, A2, A3, A4, M1, M2, M3, M4, N1, N2, N3 and N4) and healthy (sample code: HA1, HA2, HM1, HM2, HN1, HN2) plants of different citrus cultivars viz*.*, Acid lime (*Citrus aurantiifolia*), Mosambi (*Citrus sinensis*) and Nagpur mandarin (*Citrus reticulata*) were collected from different geographical regions of India and maintained in an insect-proof screen house at ICAR-CCRI, Nagpur. Four to six leaves were selected from each collected sample and washed with distilled water, wiped with 70% ethanol, blot dried and used for further processing.

### Design of CTV-specific primers and nfo-probe for RT-RPA-LFICA

The primers and probe used in the CTV-RT-RPA-LFICA were designed using Twist Amp nfo assay design manual guidelines (www.twistdx.co.uk) to amplify a segment of the coat protein gene of CTV (CTV-p25). Number of coat protein gene sequences of CTV were retrieved from the GenBank and aligned using the software MEGA7. The highly conserved region of coat protein gene (CTV isolate MD; GenBank KY011909.1) was targeted and several sets of primer combinations (4 forward and 15 reverse) were designed and custom synthesized from Integrated DNA Technologies (IDT, Iowa, USA). The standard parameters of RPA primer design were taken into consideration and in silico specificity was considered using primer-BLAST software (www.ncbi.nlm.nih.gov). After initial screening by RT-PCR and basic RPA, one optimally performing primer set (CTRPA-F1/CTRPA-R9) that consistently amplified ~ 165 bp specific region (nucleotides 237 to 402) was selected. The reverse primer (CTRPA-R9) was conjugated with the antigenic biotin molecule at the 5′ end. The corresponding TwistAmp nfo probe (nucleotides 284 to 330) (CTRPA-Probe) was designed by modifying the 31^st^ nucleotide with a base analog tetrahydrofuran (THF/dSpacer) residue and the 5ˈ termini labeled with antigenic digoxigenin molecule (Dig) whereas the 3′ termini was designed to contain a C3-spacer polymerase extension blocker. The designed probe and reverse primers were synthesized from Biosearch Technologies, USA (www.biosearchtech.com) (Table 2).

**Table 2:** RT-RPA-LFICA primers and probe used in the present study.

Sr. no	Primer/Probe Code	Sequence (5ʹ–3ʹ)	Length (mer)	Amplicon size
1	CTRPA-F1	ATAGCTATGATGTTATATCGTTTAGCGGT	29	~ 165 bp
2	CTRPA-R9-Btn	[Btn] ATTACTACAGCTACCAATAGCATTAGAG	28	
3	CTRPA-Probe	dT[DIG]-AAGTGATGATGACACCACGGGCATAACATA-dSpacer-ACTCGGGAGGGTGTT-Spacer C3 (Blocker)	46	

[Btn] = Biotin, dT[DIG] = Digoxigenin, dSpacer = Tetrahydrofuran (THF) residue.

### RNA extraction, cDNA synthesis, and conventional RT-PCR

Leaf midrib regions were minced, frozen in liquid nitrogen and 100 mg of ground powder was used for RNA isolation using the RNeasy Plant mini kit (Qiagen, Hilden, Germany) as per the manufacturer’s instruction. The concentration and quality of the extracted RNA was determined on a NanoDrop 2000 Spectrophotometer (Thermo Scientific) and stored at -80 ℃. To confirm the presence of CTV, RT-PCR was conducted using a coat protein gene specific primer pair (CN150/CN151) as previously described by Warghane et al.^43^ The PCR products were visualized by 1% agarose gel electrophoresis and the UV GelDoc system (G: Box Syngene). Another primer set, P23RBP-F/R, specific to the RNA binding protein (CTV-P23) gene located at the 3′-terminus and adjacent to the untranslated region of the RNA genome of CTV^44^ was designed and custom synthesized (IDT, Iowa, USA). It was also used to validate the RT-RPA technique (Table 3). PCR amplification was performed using the P23RBP-F/R primer set according to Kokane et al.^45^.

**Table 3:** Primer and probe sequences used for TaqMan-qPCR assay, conventional RT-PCR and generation of in vitro RNA standard in the present study.

Sr. No	Primer code	Sequence (5ʹ–3ʹ)	Length (nts)	Amplicon size
**TaqMan-qPCR assay**				
1	P25-F	AGCRGTTAAGAGTTCATCATTRC	23	~101 bp
2	P25-R	TCRGTCCAAAGTTTGTCAGA	20	
3	CTV-FAM	56-FAM/CRCCACGGGYATAACGTACACTCGG/3BHQ_1	25	
**Conventional RT-PCR**				
1	CN150	ATATATTTACTCTAGATCTACCATGGACGACGAAACAAA	39	~ 672 bp
2	CN151	GAATCGGAACGCGAATTCTCAACGTGTGTTAAATTTCC	38	
3	P23RBP-F	ATGAACGATACTAGCGGAC	19	~ 630 bp
4	P23RBP-R	GATGAAGTGGTGTTCACGG	19	
**Generation of in vitro-transcribed RNA standard**				
1	RPARNA-P25-F1	GAATTAATACGACTCACTATAGGGAGAATGGACGACGAGACGAAGAAATTG	51	~ 672 bp
2	RPARNA-P25-R2	TCAACGTGTGTTAAATTTCCC	21	

### Generation of in vitro-transcribed RNA standard for CTV-p25

To assess the sensitivity of the developed RT-RPA, an in vitro-transcribed RNA standard was generated by using a MEGAscript T7 transcription kit (Invitrogen). Total RNA was isolated from CTV infected plants and converted into single stranded cDNA using coat protein gene specific reverse primer (RPARNA-P25R2) which contains the RNA polymerase T7 promoter sequence. The cDNA was used as template for PCR amplification of the 700 bp coat protein gene using RPARNA-P25F1/R2 primers (Table 3). The PCR amplified products were checked on 1.5% agarose gel, eluted and sequenced. The sequence validated PCR product (120 ng) containing the T7 RNA polymerase promoter sequence was used as template for in vitro RNA synthesis. The in vitro RNA transcription was performed at 37 °C for 4 h in a mix consisting of 3 mM of each T7 NTPs and 2 µl Enzyme mix in 1 × T7 reaction buffer. After reaction completion, the RNA was treated with 2U TURBO DNase (2U/µl) and recovered by lithium chloride precipitation according to manufacturer's instructions. The copy number of in vitro synthesized RNA was determined using the formula, RNA copy number = Moles of ssRNA × Avogadro’s number (6.022 × 10^23^). The moles of ssRNA was calculated as mass of ssRNA (g)/[(number of ribonucleotides of ssRNA × average molecular weight of a ribonucleotide) + 18.02 g/mol].

### TaqMan-qPCR assay

TaqMan-qPCR was used to compare and validate the sensitivity of the CTV-RT-RPA-LFIC assay using the CTV specific primer–probe combination. The coat protein (CTV-p25) gene (GenBank AF260651) specific primers (forward; P25-F and reverse; P25-R) and corresponding probe (labelled with 6-carboxy-fluorescein (FAM) reporter dye at the 5′ terminus, and the Black Hole Quencher (BHQ)-1dye at the 3′ terminus) were custom synthesized at IDT^46^. Plant samples (A1, A2, A3, A4, M1, M2, M3, M4, N1, N2, N3, N4, HA1, HA2, HM1, HM2, HN1, and HN2) were examined by TaqMan-qPCR. Total RNA was converted into cDNA using coat protein gene specific reverse primer (P25R). The cDNA (1 µl) was used as a template for qPCR reactions. The primers and probe concentration were optimized for obtaining highest reporter fluorescence and the lowest Ct (Cycle threshold) value. The TaqMan-qPCR assay was performed using a StepOne Real Time PCR System (Applied Biosystems) in a total of 20 µl reaction volume containing 300 nM each of forward (P25-F) and reverse (P25-R) primers, 250 nM probe (CTV-FAM) with CXR reference dye containing 1x GoTaq qPCR master mix (Promega). The reaction protocol was 95 °C for 2 min, followed by 40 cycles at 95 °C for 15 s, annealing and primer extension for 1 min at 60 °C. All experimental reactions were conducted in triplicate along with non-template controls (NTC) and the data was analyzed using StepOne Software v2.1. To analyze the sensitivity, cDNA was synthesized from 141 ng of in vitro-transcribed RNA. The tenfold serial dilution of cDNA (1, 10^–1^, 10^–2^, 10^–3^, 10^–4^, 10^–5^, 10^–6^, 10^–7^, 10^–8^, 10^–9^ and 10^–10^) was used for detection limit estimation and validation of RT-RPA.

### Primer optimization and screening based on RT-RPA and RT-PCR

The designed sets of primer combinations (4 forward and 15 reverse) were optimized using conventional PCR and RT-RPA. The most optimally performing CTV specific primer sets were used for RT-RPA-LFICA optimization. For screening and validation of specific primers, RT-PCR was performed with different parameters which were varied depending on the primer pair. RT-RPA was also performed in a total 25 µl reaction volume that contained 1.2 μl each of forward and reverse primer (480 nM), 14.75 μl rehydration (RH) buffer, 1 μl cDNA as a template and 5.6 μl nuclease-free water. The reaction mixture was vortexed and poured into the freeze-dried reaction pellets (TwistAmp basic kit, TwistDx, Cambridge, UK) and mixed gently. To activate the reaction, 14 mM magnesium acetate (MgAc) was dispensed into the cap of the tube and spun down to start the reaction. The reaction was incubated in a dry bath (Labtech, USA) at isothermal temperature (40 °C) for 4 min, tubes were vortexed, briefly centrifuged and re-incubated for a further 21 min to achieve optimal amplification. An aliquot of 2.5 μl of SDS (from 20% stock) was added to the final RT- RPA product and heat inactivated at 65 °C for 10 min. Subsequently, the mixture was diluted with three volume of nuclease-free water, extracted with an equal volume of chloroform, and the supernatant was precipitated by chilled isopropanol at -20 °C. The precipitated product was centrifuged, washed with 70% chilled ethanol, air dried, and suspended in 30 μl of nuclease-free water and was analyzed. The in silico hetero-dimer analysis was also performed for primer and probe using IDT-OligoAnalyser tool. The reverse primer CTRPA-R1-Btn (5′-ATTAGTACGGTTACCAATACCCTTAGAGTT-3′) was gradually converted to CTRPA-R7-Btn (5′-ATTAGTACAGCTACCAATAGCATTAGAGTT-3′), CTRPA-R8-Btn (5′-ATTAGTACAGCTACCAATAGCATTAGAG-3′) and CTRPA-R9-Btn (5′-ATTACTACAGCTACCAATAGCATTAGAG-3′) by modifying nucleotides (the modified nucleotides are underlined) to optimize the complementary energy (ΔG).

### RT-RPA-Lateral flow immunochromatographic assay

The RT-RPA-LFICA was optimized using labelled primer–probe, TwistAmp nfo kit (Cambridge, UK) and PCRD nucleic acid detector, a sandwich immunochromatographic assay (Abingdon Health, UK). The 25 μl reaction was performed with 240 nM forward primer (CTRPA-F1), 240 nM reverse primer (CTRPA-R9-Btn), 40 nM probe (CTRPA-Probe), 14.75 μl RH buffer, 1 μl cDNA as a template and 6.65 μl nuclease-free water. The reaction mixture was vortexed and poured into the half of freeze-dried reaction pellets provided in the kit (TwistAmp nfo kit, Cambridge, UK) and mixed gently. To activate the reaction, 14 mM magnesium acetate (MgAc) was added into the reaction and incubated at isothermal temperature (40 °C) for 4 min, and then the tubes were vortexed, centrifuged briefly and re-incubated for a further 11 min to achieve full amplification. For LFICA analysis, the RPA product was diluted to 1:200 (0.5:100) with the PCRD extraction buffer and 75 μl of diluted reaction was loaded onto the sample pad of the PCRD nucleic acid detector and the results were observed over 5 min. The RT-RPA-LFICA was also performed at 20 °C, 25 °C, 30 °C, 35 °C, 40 °C, 42 °C, 45 °C, and 50 °C to determine the optimum temperature and the optimal reaction time was determined by performing the reaction at 0, 5, 10, 15, 20, 25 and 30 min.

### Analytical sensitivity, specificity and field validation of RT-RPA-LFICA

The sensitivity and specificity of the standardized RT-RPA-LFICA were determined using in vitro-transcribed RNA standard and other non-CTV major pathogens of citrus, respectively. In vitro-transcribed RNA and total genomic RNA of citrus infected with CTV and non CTV major pathogens were reverse transcribed to cDNA using CTV specific reverse primer. The detection limit of the assay was assessed based on the sandwich immunochromatographic assay using a tenfold serial dilution of the cDNA (1, 10^–1^, 10^–2^, 10^–3^, 10^–4^, 10^–5^, 10^–6^, 10^–7^, 10^–8^ , 10^–9^ and 10^–10^) synthesized from 141 ng of in vitro-transcribed RNA as an initial template. The cDNA dilutions were used to compare the sensitivity of the assay with RT-qPCR and RT-PCR. The sensitivity of RT-RPA-LFICA was also analyzed based on in vitro synthesized RNA as template. The RNA (235 ng/µl) was tenfold serially diluted to 23.5 ng/µl, 2.35 ng/µl, 0.235 ng/µl, 0.0235 ng/µl, 0.00235 ng/µl, 0.000235 ng/µl and 1 µl of each was used as template. All reactions were performed in triplicate and the non-template reaction mixture was included as negative control. The different samples viz*.* CTV-unknown samples, CTV positive, and other non-CTV major virus and bacterial pathogens of citrus*., Citrus yellow mosaic virus*^47,48^, *Indian citrus ringspot viru*s^20^, *Candidatus* Liberibacter asiaticus^49^, and *Candidatus* Phytoplasma cynodontis (citrus phytoplasma)^50,51^ (Supplementary Fig. S2, Table S1) were used to determine the specificity of the RT-RPA-LFICA. The developed RT-RPA-LFIC assay was validated by comparison with the TaqMan-qPCR assay, and conventional RT-PCR using known positive samples (n = 12) and (n = 6). The performance of assay for the CTV detection was determined using field samples (n = 74), which was also compared with TaqMan-qPCR and conventional RT-PCR testing. The degree of agreement between the RT-RPA-LFICA and TaqMan-qPCR assay results were measured with kappa value by using Graphpad Software (https://www.graphpad.com/quickcalcs).

## Supplementary information

Supplementary Information.

## Data Availability

The data that support the findings of this study are available from the corresponding author upon reasonable request.
