# Supplementary material for: Development of a reverse transcription recombinase polymerase based isothermal amplification coupled with lateral flow immunochromatographic assay (CTV-RT-RPA-LFICA) for rapid detection of Citrus tristeza virus
Source: Sci Rep. 2020 Nov 26;10:20593. doi: 10.1038/s41598-020-77692-w (PMC7693335; doi:10.1038/s41598-020-77692-w)
Supplement: Supplementary file 1 — Supplementary Information. [file 41598_2020_77692_MOESM1_ESM.docx]

**Development of a reverse transcription recombinase polymerase based isothermal amplification coupled with lateral flow immunochromatographic assay**

**(CTV-RT-RPA-LFICA) for rapid detection of *Citrus tristeza virus***

Dilip Kumar Ghosh^1,*^, Sunil B. Kokane^1^& Siddarame Gowda^2^

**Supplementary Figure.**


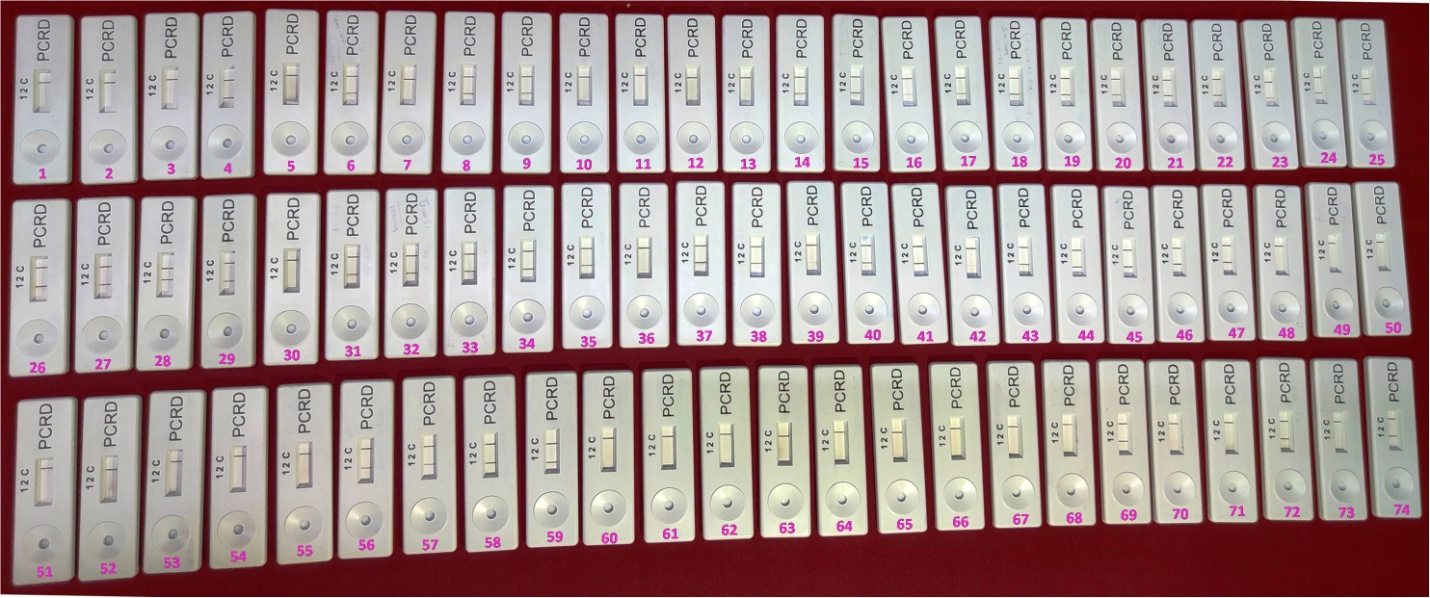


**Figure S1.** **Validation of CTV-RT-RPA-LFIC Assay using CTV suspected field samples.** Sample numbers (1-74) shown are outlined in serially in Table. 3.

**
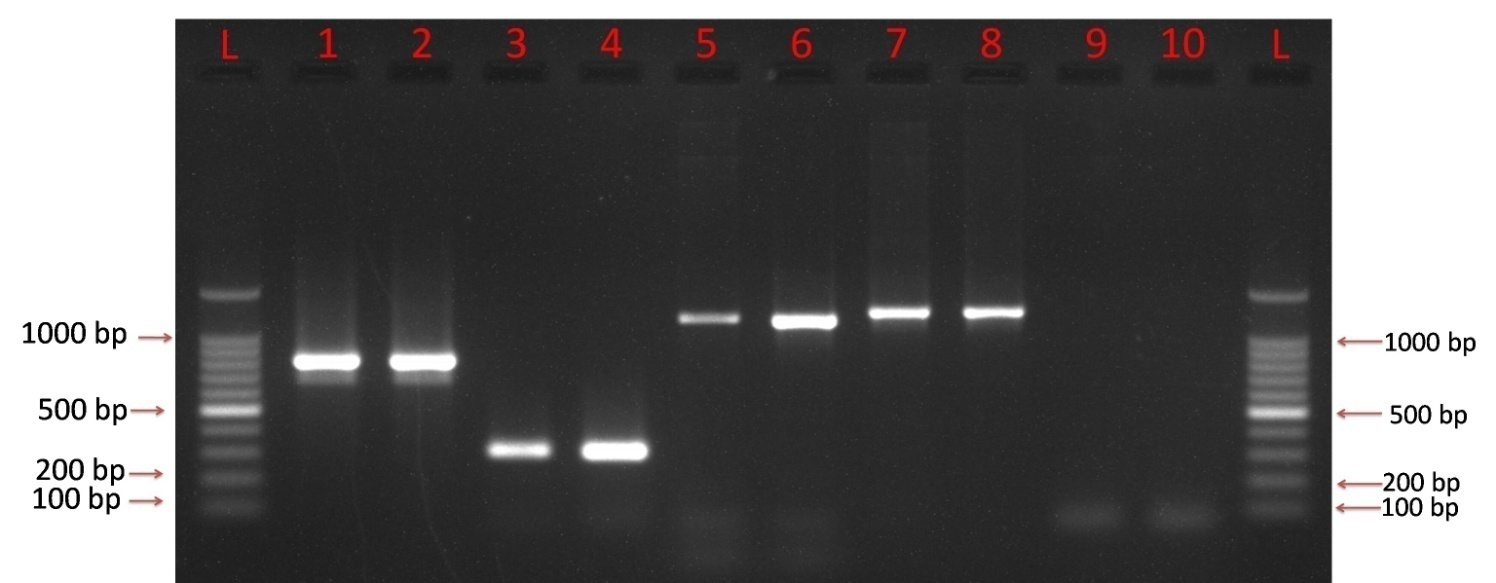
**

**Figure S2.** The amplified products of other major citrus pathogens resolved on 1.5% agarose gel electrophoresis. Lane L, 100 bp DNA Ladder; Lane 1-2, citrus yellow mosaic virus (⁓ 811 bp), Lane 3-4, Indian citrus ringspot virus (⁓309 bp), Lane 5-6, *Ca*. L. asiaticus (⁓ 1160 bp), Lane 7-8, Phytoplasma (⁓1200 bp); Lane 9, healthy plant control and Lane 10, reaction control.

**Supplementary Table.**

**Table S1.** List of primers used in detection of other major citrus pathogen

| **Sr. No** | **Primer Code** | **Sequence ( 5ʹ- 3ʹ)** | **Length (nts)** | **Target Pathogen** | **Amplicon Size (bp)** | **Reference** |
| --- | --- | --- | --- | --- | --- | --- |
| 1 | CMBV-DF1 | GATGGTTGGGGAGGTGTCTG | 20 | CYMV | ⁓811 | [47] |
| 2 | CMBV-DR1 | AGCTCGACCACTTCACACAG | 20 |  |  |  |
| 3 | RG-F | TGTTGTCCGAGACTTCTGCC | 20 | ICRSV | ⁓309 | [20] |
| 4 | RG-R | CCTTTGGTGAAGGCAACGTG | 20 |  |  |  |
| 5 | OI1 | GCGCGTATGCAATACGAGCGGCA | 23 | *Ca*. L. asiaticus | ⁓ 1160 | [52,53,54] |
| 6 | OI2c | GCCTCGCGACTTCGCAACCCAT | 22 |  |  |  |
| 7 | R16F2n | GAAACGACTGCTAAGACTGG | 20 | Phytoplasma | ⁓1200 | [50,51,55] |
| 8 | R16R2 | TGACGGGCGGTGTGTACAAACCCCG | 25 |  |  |  |
